# Editorial: Application of multi-omics analysis in thoracic cancer immunotherapy

**DOI:** 10.3389/fimmu.2024.1508723

**Published:** 2024-10-31

**Authors:** Jindong Xie, Tingting Cai, Attila Szöllősi, Yuan Li, Hailin Tang

**Affiliations:** ^1^ State Key Laboratory of Oncology in South China, Guangdong Provincial Clinical Research Center for Cancer, Sun Yat-sen University Cancer Center, Guangzhou, China; ^2^ Department of Urology, State Key Laboratory of Genetic Engineering, Collaborative Innovation Center for Genetics and Development, School of Life Sciences, Fudan University Shanghai Cancer Center, Fudan University, Shanghai, China; ^3^ Department of Immunology, Faculty of Medicine, University of Debrecen, Debrecen, Hungary; ^4^ Department of Pharmacology, School of Pharmaceutical Sciences, Cheeloo College of Medicine, Shandong University, Jinan, Shandong, China

**Keywords:** multi-omics, thoracic cancer, immunotherapy, oncology, tumor microenvironment

The advent of multi-omics analysis has fundamentally transformed our understanding of thoracic cancers, particularly in terms of their interactions with the immune system. Recent advancements in multi-omics technologies have enabled us to gain insights into tumors at the single-cell level. For instance, Spatial genomics, spatial transcriptomics, and spatial proteomics facilitate the understanding of the three-dimensional molecular architecture of tumors, elucidating the intricate interactions between tumor cells and the tumor microenvironment (TME) (1, 2). Furthermore, comprehensive analyses of circulating tumor DNA (ctDNA) and multi-omics profiling of circulating tumor cells (CTCs) provide valuable support in deciphering the dynamic changes in tumor molecular structures during progression and treatment (3).

The current Research Topic, “*The application of multi-omics analysis in thoracic cancer immunotherapy*,” brings together leading researchers in this highly anticipated field, providing a series of authoritative reviews and exciting original articles that update our understanding of immunotherapy for thoracic tumors. These studies illustrate the complexity of the TME and its impact on immune responses, underscoring the potential for personalized treatment strategies through multi-omics analysis. We anticipate that these cutting-edge studies will advance thoracic cancer immunotherapy and improve clinical outcomes for patients.

The most significant advantage of multi-omics analysis in the field of thoracic tumors and immunotherapy is its ability to comprehensively reveal the TME and its influence on immune responses. The TME consists of various non-cancerous host cells, including fibroblasts, immune cells, and endothelial cells, as well as critical components like the extracellular matrix (ECM) and soluble factors (4). In thoracic cancers, recent studies have demonstrated that these elements significantly influence tumor behavior, immune evasion, and treatment responses (5). For instance, Kang et al. revealed significant differences in the tumor microenvironment between homologous recombination deficient (HRD) and non-HRD triple-negative breast cancer samples through multi-scale transcriptomics, suggesting that combining HRD with predictive models or other immune cell content assessment methods may enhance the prediction of immunotherapy response (6). Such findings highlight the importance of characterizing the TME through multi-omics approaches.

Additionally, multi-omics analysis helps characterize the features of different tumor subtypes, facilitating the development of personalized immunotherapy strategies that enhance treatment efficacy and patient outcomes. This integrated perspective allows researchers to gain deeper insights into the biological characteristics of thoracic tumors, promoting the development of innovative therapeutic approaches. Liu et al. demonstrated that integrated multi-omics analysis of esophageal squamous cell carcinoma (ESCC) identifies four distinct subtypes, emphasizing immune response heterogeneity in chest tumors and the clinical relevance of immune modulation for better responses to immunotherapy (7).

The rapid advancement of multi-omics methods has significantly deepened our understanding of cancer’s molecular landscape. Xu et al. presented a comprehensive proteomics analysis of lung adenocarcinoma (LUAD), revealing distinct proteomic characteristics and three subtypes associated with different clinical features, thereby enhancing potential diagnosis and targeted therapy (8). Wang et al. utilized spatial transcriptomics and multiplex immunohistochemistry to reveal the molecular characteristics and cellular plasticity of distinct histologic subtypes in LUAD, highlighting the contribution of multi-omics analysis in establishing the cancer molecular landscape and identifying potential therapeutic targets for invasive LUAD (9).

However, these technologies generate vast and complex datasets, presenting a substantial challenge for translational and clinical researchers in translating intricate information into clinical outcomes that benefit cancer patients. As we navigate this complexity, innovative tumor analysis techniques hold the potential to reshape the future of precision therapy by enabling the identification of novel biomarkers, optimizing treatment strategies, and facilitating personalized medicine approaches. By effectively harnessing and interpreting multi-omics data, we can improve patient stratification, enhance treatment efficacy, and ultimately drive better clinical outcomes in cancer care.

As we look to the future, the integration of artificial intelligence (AI) and machine learning with multi-omics data holds significant promise (10). These technologies can enhance data analysis, enabling the identification of complex patterns and predictive models that inform personalized treatment strategies. By harnessing these advancements, researchers and clinicians can better tailor immunotherapy approaches to meet the unique needs of patients with thoracic cancers.

In conclusion, multi-omics analysis represents a powerful tool in the fight against thoracic cancers, particularly in enhancing our understanding of the TME and its interactions with the immune system. As we continue to unravel the complexities of cancer biology, the insights gained from multi-omics studies will be pivotal in developing more effective and personalized immunotherapeutic strategies. We look forward to the ongoing innovations in this field and their potential to improve patient outcomes in thoracic cancer treatment.
